# Breaking Down Barriers to a Suicide Prevention Helpline: Protocol for a Web-Based Randomized Controlled Trial

**DOI:** 10.2196/41078

**Published:** 2023-04-24

**Authors:** Margot C A van der Burgt, Saskia Mérelle, Willem-Paul Brinkman, Aartjan T F Beekman, Renske Gilissen

**Affiliations:** 1 Department of Research 113 Suicide Prevention Amsterdam Netherlands; 2 Department of Psychiatry Amsterdam University Medical Center Amsterdam Netherlands; 3 Department of Intelligent Systems Delft University of Technology Delft Netherlands; 4 GGZ inGeest Specialized Mental Health Care Amsterdam Netherlands

**Keywords:** barrier reduction intervention, suicidal ideation, self-help, suicide prevention helpline, randomized controlled trial, help-seeking

## Abstract

**Background:**

Globally, suicide is among the leading causes of death, with men being more at risk to die from suicide than women. Research suggests that people with suicidal ideation often struggle to find adequate help. Every month, around 4000 people fill in the anonymous self-test for suicidal thoughts on the website of the Dutch suicide prevention helpline. This self-test includes the Suicidal Ideation Attributes Scale (SIDAS), which educates users about the severity of their suicidal thoughts. The vast majority (70%) of people who complete the self-test score higher than the cutoff point (≥21) for severe suicidal thoughts. Unfortunately, despite this, less than 10% of test-takers navigate to the web page about contacting the helpline.

**Objective:**

This protocol presents the design of a web-based randomized controlled trial that aims to reduce barriers to contacting the suicide prevention helpline. The aim of this study is 2-fold: (1) to measure the effectiveness of a brief barrier reduction intervention (BRI) provided in the self-test motivating people with severe suicidal thoughts to contact the Dutch suicide prevention helpline and (2) to specifically evaluate the effectiveness of the BRI in increasing service use by high-risk groups for suicide such as men and middle-aged people.

**Methods:**

People visiting the self-test for suicidal thoughts on the website of the suicide prevention helpline will be asked to participate in a study to improve the self-test. Individuals with severe suicidal thoughts and little motivation to contact the helpline will be randomly allocated either to a brief BRI, in which they will receive a short tailored message based on their self-reported barrier to the helpline (n=388) or care as usual (general advisory text, n=388). The primary outcome measure is the use of a direct link to contact the helpline after receiving the intervention or control condition. Secondary outcomes are the self-reported likelihood of contacting the helpline (on a 5-point scale) and satisfaction with the self-test. In the BRI, participants receive tailored information to address underlying concerns and misconceptions of barriers to the helpline. A pilot study was conducted among current test-takers to identify these specific barriers.

**Results:**

The pilot study (N=1083) revealed multiple barriers to contacting the helpline. The most prominent were the belief that a conversation with a counselor would not be effective, fear of the conversation itself, and emotional concerns about talking about suicidal thoughts.

**Conclusions:**

Our study will provide insight into the effectiveness of a brief BRI designed to increase the use of a suicide prevention helpline provided in a self-test on suicidal thoughts. If successful, this intervention has the potential to be a low-cost, easily scalable, and feasible method to increase service use for helplines across the world.

**Trial Registration:**

ClinicalTrials.gov NCT05458830; https://clinicaltrials.gov/ct2/show/NCT05458830

**International Registered Report Identifier (IRRID):**

PRR1-10.2196/41078

## Introduction

According to the World Health Organization, suicide is among the leading causes of death worldwide, with more lives taken by suicide than by malaria, HIV/AIDS, breast cancer, or war and homicide. Globally, men are more likely to die from suicide than women, with a 2.3 times higher age-standardized suicide rate among males than females [1]. Unfortunately, research suggests that people with suicidal thoughts often do not seek help, making them invisible for preventive measures and targeted support [2-4]. Connecting individuals with suicidal ideation to appropriate mental health care services is, therefore, a key preventive effort.

A recently conducted systematic review by Tang et al [5] examined the factors associated with not using formal mental health services among individuals who died by suicide. They found the following key factors: male sex, both younger and older age, and living in a rural location. A subsequent systematic review by the same authors identified predictors of not receiving formal mental health services among people at risk of suicide. The findings of this review indicated that among people experiencing suicidality, minority ethnicity, better perceived general health, lower psychological distress, lower severity of suicidality, no mental health diagnosis, a lower perceived need for treatment, and lower use of medical services are all associated with nonreceipt of formal mental health services [6].

It is also assumed that a considerable percentage of people with suicidal ideation do not disclose their ideation. Studies among the general public in France and the Netherlands indicate that almost half of the adults with suicidal ideation do not disclose their thoughts about suicide to others [7,8]. And even when people are engaged in mental health care, disclosure of suicidality is not self-evident. A recent study among young Australians (aged 16-25 years) who had experienced suicidal ideation and were engaged with a mental health professional, revealed that 39% had never disclosed their suicidality to their clinician, with concerns about confidentiality as the most common reason [9].

In order to increase help-seeking behavior, it is important to have insight into the barriers that people experience when seeking help for suicidal thoughts. Known barriers to care are a lack of perceived need for services, the preference for self-management, fear of hospitalization, structural factors like time and finances, and stigmatizing attitudes toward suicide, mental health problems, and toward seeking professional treatment [3,10]. Another important issue is that of unmet mental health needs. Individuals may not receive the help they need because there are insufficient services available, they are unable to afford the cost of care, or they feel their treatment is not meeting their needs [11,12].

Previous studies that compared web-based and offline help-seeking among people with suicidal ideation found that people who sought help on the internet reported higher levels of suicidal ideation, indicating that individuals may choose to go on the internet when their suicidality becomes more severe [13,14]. Anonymous digital help can be a low-threshold first step toward professional face-to-face help. In their innovative study, Jaroszewski et al [15] provide evidence that a brief, risk assessment and intervention platform increased crisis service use among those on the internet and in current mental health crisis. Due to their accessible, affordable, and often anonymous nature, digital innovations have the potential to reduce structural barriers to help-seeking. But as Jaroszweski et al [15] state, attitudinal barriers like the preference for informal help remain. It is, therefore, important to develop and evaluate interventions that address those barriers, particularly on digital platforms. However, robust research on reducing barriers to help-seeking in a web-based environment is scarce.

In the Netherlands, the suicide prevention organization “113 Suicide Prevention” provides around-the-clock anonymous support by phone and chat, as well as a web-based self-help course, self-assessment tests, and brief web-based coaching and therapy. Since its opening in 2009, the organization has seen an annual increase in brand awareness and service users, with almost 140,000 chat and phone call conversations and 1.3 million website visits in 2021 [16]. Apart from the homepage, the “test yourself” page is the most visited section of the helpline’s website. Every month, around 4000 people fill in the anonymous self-test for suicidal thoughts. This self-test includes the Suicidal Ideation Attributes Scale (SIDAS) and informs the test-taker of the severity of their suicidal thoughts [17]. Although the vast majority (70%) of people who complete the test score higher than the cutoff point (≥21) for severe suicidal thoughts, only approximately 10% of test-takers go on to navigate to the web page about contacting the helpline. And although due to the anonymity of the helpline’s services, it is not possible to know if people follow up on the advice of contacting the helpline, the difference in demographic distributions between the users of the self-test and the crisis helpline confirms the assumption that a substantial group does not continue using helpline service. While the percentage of men among the self-test users is 40%, it is only around 25% among the users of the helpline [18].

This paper presents the design of a web-based randomized controlled trial (RCT) that aims to reduce barriers to a suicide prevention helpline. The trial is inspired by Jaroszewski et al’s [15] study in which they evaluated a brief, automated barrier reduction intervention (BRI) designed to increase the use of crisis service referrals provided in the mental health app, Koko [19]. Although help-seeking behavior and barriers have been studied in traditional mental health care, guiding reluctant high-risk individuals in web-based environments toward professional help is still relatively uncharted territory. To the best of our knowledge, this study will be the first web-based RCT among people with severe suicidal ideation aimed to reduce the barriers to a helpline. The aim of this study is 2-fold: (1) to measure the effectiveness of a brief barrier reduction intervention (BRI) provided in the self-test motivating people with severe suicidal thoughts to contact the Dutch suicide prevention helpline and (2) to specifically evaluate the effectiveness of the BRI in increasing service use by high-risk groups for suicide such as men and middle-aged people [20].

## Methods

### Study Design

The study is designed as a web-based randomized controlled trial for the anonymous users of a web-based self-test for suicidal thoughts in which individuals with severe suicidal thoughts and no interest in contacting the helpline will be randomly allocated either to a short BRI or receive a general advisory text (care as usual). To minimize the burden on our high-risk and sensitive study population, we aim that it is feasible to complete the intervention within 10 minutes.

### Participants

The participants of the trial will be recruited among the anonymous visitors to the helpline’s website. People who would like to participate will be directed to a web-based information letter and consent form. If they give their consent to the processing of their data for research purposes, the participants will be transferred to the web-based RCT on the survey platform Qualtrics. To assure strict anonymity, no identifying information or IP addresses will be collected.

### Exclusion Criteria

Participants will be excluded from the study if (1) they are younger than 16 years old, (2) they score below the cutoff point for severe suicidal thoughts (SIDAS score <21), (3) they score above the cutoff point for severe suicidal thoughts (SIDAS score ≥21) and report being likely to contact the suicide prevention helpline. They will be directly transferred to the contact details of the helpline.

People who do not meet the inclusion criteria will be directed to a web page thanking them for their time and encouraging them to contact the helpline if they feel distressed.

### Sample Size

When assuming probabilities of 0.49 and 0.39 for contacting the helpline for the intervention group and the control group, respectively (based on Jaroszweski et al [15]), a power of .8 and alpha of .05, a sample size calculation for logistical regression analysis (2-tailed) indicates a total sample size of 775 participants [21]. We estimate that about 10% of participants drop out during the intervention; therefore, at least 853 participants have to be included. Furthermore, we estimate that approximately 30% of respondents score below the cutoff point for high risk of suicidal behavior, and based on Jaroszewski et al [15], we expect that 20% of respondents will indicate a high probability of contacting the helpline [15]. We, therefore, estimate that a minimum of 1706 respondents need to be recruited. We will continue to recruit participants until there are enough participants per condition.

### Procedure

**Figure 1:**
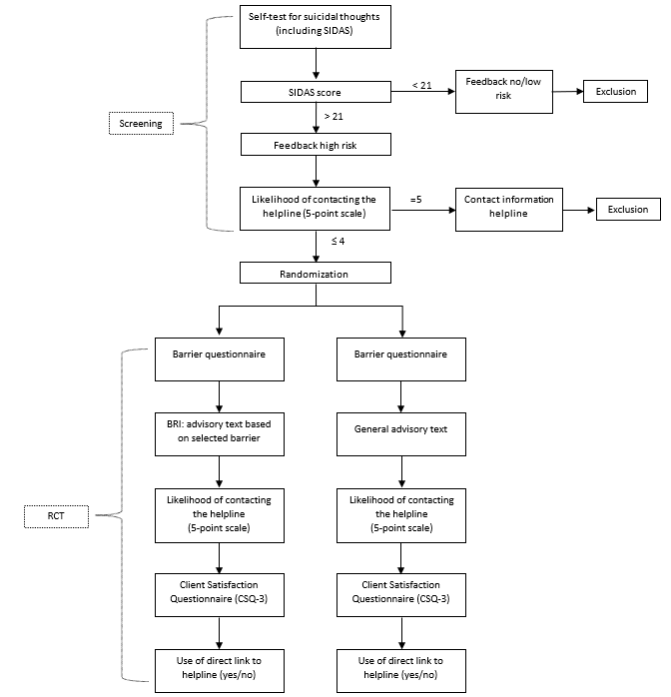
Flowchart. BRI: barrier reduction intervention; RCT: randomized controlled trial; SIDAS: Suicidal Ideation Attributes Scale.

After giving their informed consent and stating that they are 16 years or older, participants will be transferred from the helpline’s website to the Qualtrics platform. Figure 1 displays the study’s flowchart. During the screening phase, respondents start with the self-test. The self-test includes the SIDAS, which consists of 5 items on a 10-point scale measuring the frequency of suicidal thoughts, controllability, closeness to an attempt, distress, and interference with daily activities [17]. The self-test also contains questions regarding gender, age, and if the test-taker is currently in treatment for mental health problems. As mentioned in the exclusion criteria, if respondents score below the cutoff point for severe suicidal thoughts or score above the cutoff point but report that they are likely to contact the helpline, they will be excluded from the RCT.

After inclusion, participants will be randomly assigned to either the intervention or control condition. In both conditions, participants receive a barrier questionnaire to identify the reason why their self-reported likelihood of contacting the helpline in the screening phase was not very likely. After the barrier questionnaire, they receive a general advisory text (control) or an advisory text based on their selected barrier.

### Measures

The primary outcome measure is the use of a direct link to the helpline after completing the intervention or the control condition. Due to the anonymous nature of the helpline, it is not possible to measure if people who do not directly use the link to the helpline, contact the helpline at a later point in time. Therefore, the self-reported likelihood of contacting the helpline will be included as a proxy measurement. This will be measured by the question “How likely are you to contact 113's helpline via chat or phone?” with answering options ranging from “not likely” to “very likely” on a 5-point scale. Other measures include gender, age group, SIDAS score, being in treatment for mental health problems (yes, no, and on a waiting list), and satisfaction with the self-test (Dutch CSQ-3) [22].

### Persuasive System Design Model

Preceding research and literature on the design of persuasive eHealth technologies often cover software or applications intended for continuous use, whereas our study entails a single-session intervention. With this in mind, the BRI has been designed with the use of the persuasive system design (PSD) model [23,24]. The PSD model is a framework for the development and evaluation of persuasive behavior change technologies and offers 28 design principles [23]. Table 1 gives an overview of the applicable and relevant principles for our intervention, the corresponding requirements, and the implementation goals for the BRI.

**Table 1:** Design principles originating from the persuasive system design model applied to the intervention.

Principle [24]			Requirement		Implementation goals
**Primary task support**					
	Reduction (“A system that reduces complex behavior into simple tasks”)	The system should lower the barrier toward help-seeking; breaking it down into small steps.		Guiding toward the first small step in the help-seeking process: emphasis on different low-threshold methods of contact (24/7 chat and telephone) and the anonymous nature of the helpline	
	Tunneling (“Using the system to guide users through a process or experience provides opportunities to persuade along the way”)	The system should guide users in the attitude change process by providing means for action.		Self-test offers the test-taker information about the severity of their suicidal thoughts.	
	Tailoring (“Information provided by the system will be more persuasive if it is tailored to the potential needs, interests, personality, usage context, or other factors relevant to a user group”)	The system should provide tailored information based on the user’s level of suicidality and barriers toward the helpline.		User receives feedback on their level of suicidality. BRI^a^ provides different information based on the selected barrier.	
	Simulation (“Systems that provide simulations can persuade by enabling users to observe immediately the link between cause and effect”)	The system should persuade users to contact the helpline with experiences from others.		BRI provides statements of people with lived experiences of suicidality.	
**Dialogue support**					
	Similarity (“People are more readily persuaded through systems that remind them of themselves in some meaningful way”)	The system should be designed in such a way that users can identify with the stories.		BRI includes photos from people with lived experience and counselors of the helpline of different ages and gender.	
	Liking (“A system that is visually attractive for its users is likely more persuasive”)	The system should appeal to the target group.		Self-test and BRI use the same design and tone of voice as the helpline’s website.	
**System credibility support**					
	Trustworthiness (“A system that is viewed as trustworthy will have increased powers of persuasion”)	The system should provide information that is truthful, genuine, and unbiased.		Feedback after the self-test score and information offered in BRI are written in a noncoercive style and leave all autonomy to the user, for example, “We respect your decision, of course, but would encourage you to talk about your feelings, either with us or with someone close to you.”	
	Expertise (“A system that is viewed as incorporating expertise will have increased powers of persuasion”)	The system should provide information that conveys knowledge, experience, and competence.		The BRI was built on the results of a pilot study conducted among the users of the current self-test. The information or feedback provided has been developed by the professionals of 113 (psychologists, communication advisors, and researchers).	
	Real-world feel (“A system that highlights people or organizations behind its content or services will have more credibility”)	The system should provide information about the organization and the people behind it.		BRI includes photos and experiences from counselors telling about their work in the helpline.	
	Authority (“A system that leverages roles of authority will have enhanced powers of persuasion”)	The system should convey authority.		Self-test and BRI are in the same style and tone of voice as the helpline’s website and include the helpline’s logo. BRI includes persuasive messages about the many help seekers that contact the helpline. BRI includes persuasive messages about the expertise of the helpline and its counselors.	

^a^BRI: barrier reduction intervention.

### Tailored Information

After selecting a barrier in the barrier survey, participants receive tailored information with the purpose of addressing common concerns and misconceptions about the helpline. Tailored information is useful for giving people the right information and suitable feedback and helps with selecting the most effective persuasive strategies. To persuade people to overcome their barriers to the helpline, the principles of the PSD model as well as the principles of consistency, social proof, likability, and authority derived from Cialdini’s [24] principles of influence will be used [23].

### Statistical Analysis

The analyses will be carried out with an intention-to-treat analysis. Missing values will be imputed using the MICE package in R (v4.2.2; R Core Team 2022) [25]. In order to verify the randomization process and inspect drop-outs, independent *t* tests and chi-square tests will be used to determine whether the control group and the intervention group, as well as the completers and noncompleters, are comparable on baseline factors (ie, gender, age, SIDAS score, and in treatment or not). Chi-square analysis will also be used to test the hypothesis that participants who receive the brief BRI are more motivated to contact the suicide prevention helpline (using the direct link yes/no) than participants in the control condition. To evaluate the effectivity of the BRI in increasing service use by men and middle-aged people, and to examine potential moderators, logistic regression analysis will be used.

### Ethics Approval

This study protocol is designed in accordance with the relevant guidelines. This research is not subject to the Research Involving Human Subjects Act (Wet medisch-wetenschappelijk onderzoek met mensen) because participants are not subject to procedures and are not required to follow rules of behavior. All participants of the trial will be directed to a web-based information letter and consent form. After giving consent and stating to be 16 years or older, respondents will be transferred to the web-based RCT. No identifying information or IP addresses will be gathered to ensure strict anonymity. This study was reviewed and approved by the Medical Ethics Committee of the Vrije Universiteit Medical Centre (registration number 2021.0443) and is registered at ClinicalTrials.gov (NCT05458830).

## Results

### Overview

The BRI has been designed with the use of the PSD model, the expertise of the professionals at the helpline, and relevant literature. For the design of the barrier questionnaire, a pilot study was carried out among the users of the current self-test.

### Pilot Study

To gain insight into the specific barriers to the helpline of people who fill in the self-test, a pilot study has been carried out. Between August 5 and October 14, 2021, people who took the self-test were asked the following additional questions; “What is the likelihood of you contacting the helpline to talk to our counsellors about your thoughts about suicide?” (5-point scale) and “If this likelihood is small, would you please indicate the main reason why you do not want to talk to one of our counsellors at the moment?” (open text field). During the pilot study, 7252 people filled in the self-test, of which 72% (n=5200) scored higher than the cutoff point (≥21) for severe suicidal thoughts. Almost 20% (n=1441) of all test-takers completed the open question, with 82% (n=1175) of them scoring above the cutoff point for severe suicidal thoughts. Cleaning the data led to a data set containing the answers of 1083 people in the high-risk category. Their answers revealed multiple barriers to contacting the helpline. The most prominent of them was mentioned 259 times and was the belief that a conversation with a counselor would not be effective (eg, “nobody can help me,” “it has no point anyway,” “I don’t see any possibility that my situation will change for the better,” “I already had professional help before and nothing helps,” “they would not believe me and think I exaggerate”). The second most mentioned barrier (151 times) concerned the fear of the conversation itself (eg, “I’m afraid to call,” “I’m too nervous and scared,” “I’m afraid to talk to people I don’t know,” “I don’t know what to expect”). The third most mentioned barrier (148 times) can be described as emotional concerns regarding talking about suicidal ideation (eg, “I’m not ready to talk about it yet,” “I’m afraid to talk about those feelings,” “I’m afraid that when I talk about it out loud it will get worse,” “I would not know what to say,” “I cannot articulate my feelings”). The fourth most frequently observed barrier concerned shame, stigma, and the fear that someone finds out about their contact with the helpline (eg, “I’m ashamed of my feelings,” “I’m afraid my parents will find out,” “I don’t want my spouse to know”). Other barriers concerned the topics of not wanting to be helped, being afraid to be a burden to someone, believing their problems were not bad enough, precious negative experiences with the helpline, wanting to solve their problem themselves, being already in treatment, having no energy or motivation, being afraid of the consequences (eg, having to go to a facility/crisis help or police involvement), or practical reasons such as not being in the right environment or having time restrictions.

### Assessment of Barrier

To be able to provide customized information and advice, a barrier survey is included in the BRI. To not overwhelm the test-takers or expose them to more barriers than they have thought of themselves, the barrier questionnaire lists a limited number of options. As described earlier, previous studies identified the following key barriers to care: lack of perceived need for services; the preference for self-management; the fear of hospitalization; structural factors (eg, time and finances); and stigmatizing attitudes toward suicide, mental health problems, and seeking professional treatment [3,10]. Although these barriers were reflected among the users of the self-test, the pilot study revealed other, more frequently mentioned, barriers among our target population. We, therefore, chose to include the following barriers in the BRI: (1) “I don’t think that 113 can help me,” (2) “I’m scared to talk about my feelings,” (3) “I don’t think that my problems are serious enough for 113,” (4) “I’m scared that people will find out,” (5) “I would rather solve it myself,” and the remaining option (6) “I have other reasons.”

### Testing of Prototypes

**Figure 2:**
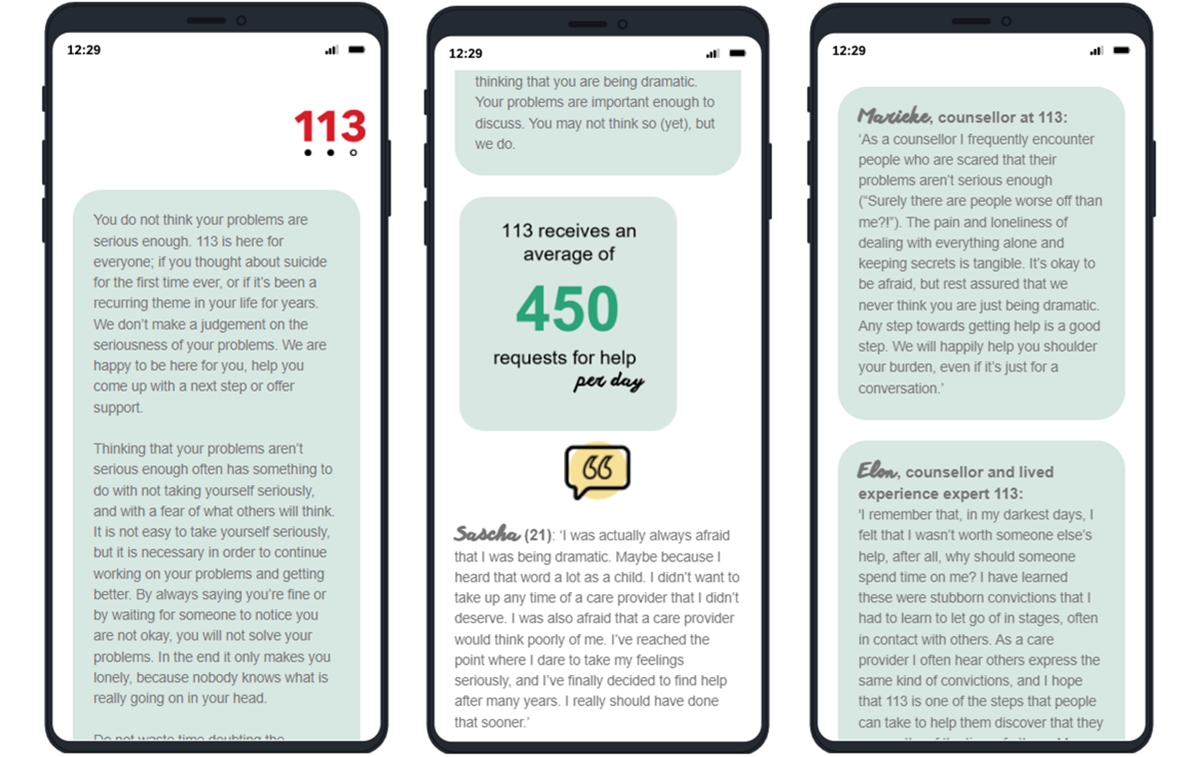
Screenshots of barrier reduction intervention. Note: translated from Dutch to English.

During the testing phase of the prototype, which included the names and photos of some counselors, the staff raised concerns regarding the anonymity and privacy of the counselors. We, therefore, decided to design the brief BRI with text-based feedback only and to anonymize the participating counselors (see Figure 2).

## Discussion

### Principal Findings

This paper describes the study protocol for a web-based RCT of a brief barrier reduction intervention. The aim of the trial is 2-fold: (1) to measure the effectiveness of a brief BRI provided in the self-test motivating people with severe suicidal thoughts to contact the Dutch suicide prevention helpline and (2) to specifically evaluate the effectivity of the BRI in increasing service use by high-risk groups such as men and middle-aged people. The goal of the intervention is to reduce self-stigma, provide information, and clear up misconceptions on which barriers to the helpline are based. To identify these barriers specific to self-test users, a pilot study was conducted. The open-ended answers of 1083 current self-test users in the high-risk category revealed multiple barriers to the helpline, of which the most common were the belief that a conversation with a counselor would not be effective, fear of the conversation itself, and emotional concerns about talking about suicidal thoughts. These identified barriers were used to design the barrier questionnaire.

### Limitations

To our best knowledge, our study will be the first web-based RCT among people with severe suicidal ideation aimed to reduce the barriers to a suicide prevention helpline. One of the strengths of the study that should not be underestimated is the fact that it will take place among the actual service users of 113 Suicide Prevention, a difficult-to-reach and high-risk population. Moreover, by measuring the intended outcome measure, contacting the helpline, behavioral as well as attitudinal, we increase the measure’s reliability and validity. Furthermore, we expect to achieve a substantial sample size and will, therefore, be able to provide a reliable estimate of the intervention’s effectiveness. However, our study is not without limitations. First, although the study takes place among the target population, it is still possible that some form of selection bias will occur as it is less likely that users in severe distress will participate in the study. Second, as mentioned before, due to the helpline’s anonymous nature it is not possible to have a follow-up measurement to determine if those who will not use the link directly after the intervention will contact the helpline at a later moment in time. For that reason, a respondent’s self-reported likelihood of contacting the helpline will be measured as well as the use of a direct link to the helpline. Third, the BRI is only text-based, it would also be valuable to examine the effects of different components (eg, video material) in a BRI.

### Conclusions

The short barrier reduction intervention built in a self-test for suicidal thoughts aims to motivate people with severe suicidal thoughts to contact the Dutch suicide prevention helpline. Guiding people with suicidal ideation to appropriate resources is a key aspect of suicide prevention. Our study will add to a growing body of research on web-based mental health interventions and provide a better understanding of the effect of tailored information and dispelling misconceptions in a high-risk group. Furthermore, if it proves to be effective, this intervention has the potential to be a low-cost, highly scalable, and easily implementable method to increase service use for mental health and crisis helplines worldwide.

## Abbreviations

BRI: barrier reduction intervention
PSD: persuasive system design
RCT: randomized controlled trial
SIDAS: Suicidal Ideation Attributes Scale

## Data Availability

The data set generated and analyzed during this study will not be publicly available due to company policies regarding help-seekers’ data but are available from the corresponding author upon reasonable request.
